# General practitioners may improve cervical screening equality in France

**DOI:** 10.1186/s12889-024-18942-8

**Published:** 2024-10-09

**Authors:** Vladimir Druel, Cyrille Delpierre, Lisa Ouanhnon, Marie-Eve Rougé Bugat, Pascale Grosclaude

**Affiliations:** 1 Département Universitaire de Médecine Générale, Université de Toulouse, Faculté de Médecine, Toulouse, 31062 France; 2 Equity Team: Team Labeled By the French League Against Cancer, UMR1295 CERPOP, Toulouse, 31073 France; 3 Registre des cancers du Tarn, Institut Universitaire du Cancer de Toulouse-Oncopole (Institut Claudius Regaud), Toulouse, 31059 France

**Keywords:** Screening, Cervical cancer, Public health, Social inequalities, Health care access

## Abstract

**Background:**

Vulnerable social groups have greater difficulty in accessing care and a lower quality of care. Health systems focused on primary care appear to be more effective, efficient and equitable. However, difficulties in accessing primary care are persisting*.* We focused on primary care screening for cervical cancer through Cervico-Uterine Smear (CUS), which has been shown to be effective in reducing disease incidence and mortality. In this study, we aimed to investigate the characteristics of women who undergo CUS according to the category of health professionals (general practitioners or gynaecologists) performing CUS and to analyse potential differences in access to care in terms of socioeconomic and geographical characteristics.

**Methods:**

This was a retrospective observational study based on data from the main health insurance schemes in France, allowing analysis of health care consumption according to socioeconomic levels and proximity to health care services. We included women aged 25 to 64 years in 2012 for whom CUS would be a relevant procedure (695,694). The sociodemographic and territorial indicators were age, geographical area deprivation, and the availability of gynaecological care. The analysis was performed using multinomial logistic regression.

**Results:**

A total of 202,271 (29%) patients underwent CUS; of whom 68% underwent CUS administered by gynaecologists and 28% were administered by general practitioners (GPs). However, inequalities in CUS screening rates were observed, with a decrease in the number of CUSs performed with increased age, a rural location, deprivation, and sparse health care provisions. Deprived people seemed less penalised by GPs.

**Conclusions:**

Involvement of General Practitioners may improve cervical screening equality in France. The organisation of health systems around primary care may allow a better access to care and to account for the specific needs of deprived populations.

**Supplementary Information:**

The online version contains supplementary material available at 10.1186/s12889-024-18942-8.

## Background

“General practitioners provide ‘first’ medical care for all categories of patients; and they provide care that is continuous over time” [1]. Primary care as a whole acts to coordinate with other specialists the patient may need [1]. Health systems focused on primary care tends to be more effective, efficient and equitable than those dominated by specialists [2]. Several studies have demonstrated that when access to primary care is ensured, inequalities in care are reduced, quality of care is improved, and overall mortality is reduced [2–6]. However, in 2016, a survey involving 11 Western countries (response rate 11–47%) showed that one in five adults (21%) had difficulty accessing primary care (18% in France) [7]. In addition, even when healthcare access is guaranteed, the quality of care received by patients may be unequal, particularly in relation with a lack of coordination [8]. The inequalities in access to health care services are often linked to the socioeconomic, geographical, cultural or even ethnic characteristics of patients [7]. Facilitating access to GPs, i.e., primary care physicians, would improve the health care of the general population [2].

Screening is part of primary or first-line care [9]. Cervical cancer screening through Cervico-Uterine Smear (CUS) has been shown to be effective at reducing mortality and the incidence of cervical cancer [10]. The national recommendations published in France in 2012 advised that CUS should be performed every three years, for women between 25 and 64 years old, if two previous CUSs performed one year apart had demonstrated normal results [11]. These national recommendations defined by the French National Authority for Health (Haute Autorité de Santé) apply to all healthcare professionals throughout France. They were modified in 2021 with the addition of papilloma virus screening after the age of 30, followed by a vaginal HPV test alone (without systematic CUS) every 5 years. All the tests could be performed by any competent professional, mainly gynaecologists, GPs or midwives.

A study of health care consumption in 2012 in Midi-Pyrénées, a region in southwestern France with a population of nearly 3 million, showed significant inequalities in access to care according to patient deprivation [12]. People living in most deprived areas had less access to GPs, gynaecologists, and related care, including CUS.

We investigated the characteristics of women who undergo CUS according to the category of health professionals (general practitioners or gynaecologists) performing CUS. The aim was to analyse potential differences between women who consult gynaecologists or general practitioners, particularly in terms of socioeconomic level and geographical area of residence.

## Methods

The database was compiled using data from the three main health insurance schemes in France. Health insurance beneficiaries living in the Midi-Pyrénées area as of December 31, 2012 (2,574,310 people, i.e., 87% of the population of Midi-Pyrénées) were included. Data on health care consumption from 1 January to 31 December 2012 were collected. The database and its construction have been described in detail elsewhere [13]. We selected all women aged 25–64 years, i.e., the target age for CUS [11], i.e., 695 694 women.

### Outcome

Gynaecological follow-up was studied in women aged 25 to 64 years [11]. For women who received CUS, we considered that the smear had been taken by the gynaecologist if the woman had consulted him or her at least once during the year. If she had not consulted a gynaecologist but a GP, we considered that the smear had been performed by the GP. Some women had a history of CUS without a gynaecologist or GP; they were not studied further in this study. In this work, women were dichotomized between those with at least one smear during the year and those without any smear.

### Covariables

Age (5-year age groups), address of residence, and benefit of the universal supplementary health coverage (CMU-C, Couverture Maladie Universelle Complémentaire) were collected. The CMU-C is granted on an income- and composition-assessed basis to households to cover the entire costs of their medical care. The CMU-C can be considered a marker of a very low socioeconomic individual level since it can be obtained for an income equivalent to approximately 62% of the French minimum income for a single person in 2019 [13]. In 2017, 5.5 million people benefited from the CMU-C, representing 8.2% of the French population [14].

Three variables were computed from the address of residence: an indicator reflecting the level of deprivation (EDI, European Deprivation Index), an indicator of the availability of gynaecological care (APL, Accessibilité Potentielle Localisée) and a variable reflecting the level of urbanisation (Toulouse agglomeration/ urban/rural areas).

The European Deprivation Index (EDI) [15] is an ecological index. It measures deprivation, defined as "a demonstrable state of deprivation relative to the local community or wider society to which a person belongs" [16]. The EDI has been used to compensate for the lack of individual socioeconomic data [15, 17] but does not allow the contextual and/or neighbourhood effect to be included. Indices based on census data use area-of-residence variables to obtain an ecological index of deprivation, which is used as a proxy for measuring individual deprivation. A high EDI value indicates a high level of deprivation. We used deciles relative to the study population. A variable CMU-C*EDI was defined to identify the most deprived patients according to the benefit of CMU-C and within the geographical zones of deprivation defined by the EDI. This allowed us to evaluate the composition of this population according to the EDI.

The APL in gynaecology was developed in France by the DRESS (Direction de la Recherche, des Etudes, de l'évaluation et des Statistiques) [18]. It measures the possibility of accessing health professionals—here, accessibility to the gynaecologist. The availability of health care professionals should be considered when defining the specific health care offered. The survey also included the needs of inhabitants by differentiating the rate of recourse to health professionals according to the age of the inhabitants. The APL also considers the availability and demand for care in the surrounding areas. It was expressed in terms of the number of full-time equivalent employees per 100,000 inhabitants. The APL was divided into deciles according to the population of women aged 25 to 64 years in our sample.

We created a variable according to three geographical areas based on the 2010 urban areas identified by the National Institute of Statistics and Economic Studies in France (INSEE) [19]:

- The Toulouse agglomeration, which included areas with more than 10,000 employees and their surrounding areas in the department;
- Urban areas, which included areas with more than 10,000 people and surrounding areas outside the Toulouse conurbation area;
- Areas with fewer than 10,000 employees were considered rural.

### Statistical analysis

The bivariate analysis results were presented as percentages and odds ratios according to the use of CUS. The unadjusted and adjusted odds ratios were calculated using multinomial logistic regression models to study the probability of receiving CUS from a GP or a gynaecologist versus not receiving it. In a second step, we compared the populations with the lowest and highest supply of gynaecological care to study whether performing CUS depended on the supply of care. During the multivariate analyses, the p-value was modified with the Holm correction to limit the risk of type I error.

Statistical analyses were conducted using the free software R and its interface R studio Version 1.1.456—© 2009–2018.

## Results

**Fig. 1:**
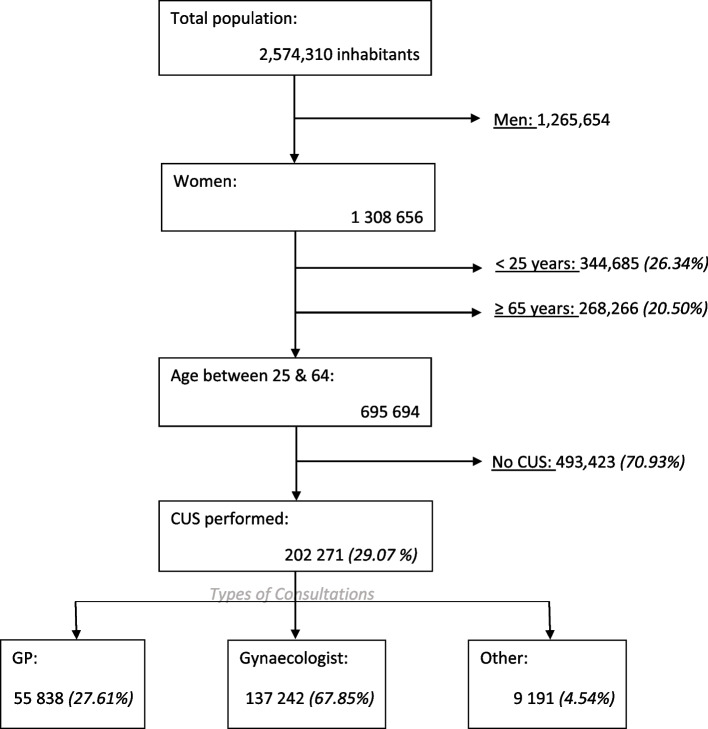
Study population and events studied (the 2012 Midi-Pyrenees population)

In 2012, 202,271 women (29.1%) received CUS (Fig. 1). The probability of not receiving CUS was greater for older women (median age 45.9 years versus 42.9 years, ranging from 33.2% at 30–35 years to 20.5% at 60–64 years), those living in a rural area, those receiving CMU-c, and those living in the most deprived areas (high EDI) (Table 1). Women living in the most deprived areas received fewer CUS, regardless of their CMU-C status. Patients who benefited from CMU-C were less likely to receive CUS. The increase in the availability of gynaecology services measured by the APL was associated with an increase in the uptake of CUS (Table 1).

**Table 1:** Characteristics of the women included according to the practitioner performing the CUS

		**CUS performed**				**No CUS**	**Total**	
		**Total**	**by GP**	**by Gynaeco**	**by Others**			
		*%*	*%*	*%*	*%*	*%*	Nb	*(%)*
		*29.07%*	*8.03%*	*19.73%*	*1.32%*	*70.93%*	695 694	*100%*
**Age** **[5 years]**	25–29	31.30%	*7.05%*	*22.29%*	*1.96%*	*68.70%*	82 413	*11.85%*
	30–34	33.22%	*7.75%*	*23.91%*	*1.57%*	*66.78%*	88 249	*12.69%*
	35–39	32.92%	*8.73%*	*22.67%*	*1.52%*	*67.08%*	85 200	*12.25%*
	40–44	32.19%	*9.26%*	*21.49%*	*1.43%*	*67.81%*	92 964	*13.36%*
	45–49	31.20%	*8.92%*	*20.86%*	*1.42%*	*68.80%*	94 291	*13.55%*
	50–54	27.31%	*8.09%*	*18.07%*	*1.15%*	*72.69%*	88 241	*12.68%*
	55–59	22.86%	*7.29%*	*14.77%*	*0.80%*	*77.14%*	83 127	*11.95%*
	60–64	20.52%	*6.81%*	*13.04%*	*0.67%*	*79.48%*	81 209	*11.67%*
**Rural/urban areas**	Toulouse	32.56%	7.88%	23.36%	1.32%	67.44%	299 532	43.06%
	Urban	28.32%	8.20%	18.72%	1.40%	71.68%	173 492	24.94%
	Rural	24.98%	8.09%	15.63%	1.26%	75.02%	222 670	32.01%
**No** **CMU**	EDI 1 privileged	34.95%	8.60%	24.91%	1.43%	65.05%	59399	8.54%
	EDI 2	33.50%	8.61%	23.48%	1.41%	66.50%	66899	9.62%
	EDI 3	31.98%	9.26%	21.38%	1.35%	68.02%	56975	8.19%
	EDI 4	30.90%	8.53%	21.01%	1.37%	69.10%	56198	8.08%
	EDI 5	29.74%	8.23%	20.22%	1.30%	70.26%	60004	8.63%
	EDI 6	28.39%	8.17%	18.85%	1.38%	71.61%	67174	9.66%
	EDI 7	28.65%	7.88%	19.36%	1.42%	71.35%	65475	9.41%
	EDI 8	28.11%	7.88%	18.79%	1.44%	71.89%	62924	9.04%
	EDI 9	27.06%	7.29%	18.36%	1.40%	72.94%	71460	10.27%
	EDI 10 deprived	24.73%	6.79%	16.69%	1.25%	75.27%	73732	10.60%
**CMU**	EDI 1 privileged	26.15%	8.60%	16.26%	1.29%	73.85%	1476	0.21%
	EDI 2	23.74%	7.02%	16.01%	0.71%	76.26%	2536	0.36%
	EDI 3	24.14%	8.89%	14.40%	0.84%	75.86%	2486	0.36%
	EDI 4	23.16%	7.58%	14.85%	0.74%	76.84%	2970	0.43%
	EDI 5	23.78%	8.37%	14.51%	0.91%	76.22%	3646	0.52%
	EDI 6	21.57%	7.40%	13.70%	0.47%	78.43%	4446	0.64%
	EDI 7	22.02%	7.02%	14.30%	0.70%	77.98%	4999	0.72%
	EDI 8	23.44%	7.83%	14.66%	0.95%	76.56%	5874	0.84%
	EDI 9	21.82%	7.11%	13.97%	0.73%	78.18%	8888	1.28%
	EDI 10 deprived	22.36%	7.29%	14.48%	0.60%	77.64%	18133	2.61%
**APL Gyn**	1 Low availability	23.06%	*7.68%*	*14.13%*	*1.25%*	*76.94%*	69 475	*9.99%*
	2	26.04%	*8.41%*	*16.47%*	*1.16%*	*73.96%*	69 899	*10.05%*
	3	27.26%	*8.29%*	*17.72%*	*1.26%*	*72.74%*	67 578	*9.71%*
	4	29.05%	*8.47%*	*19.32%*	*1.27%*	*70.95%*	71 328	*10.25%*
	5	31.41%	*8.83%*	*21.26%*	*1.32%*	*68.59%*	69 943	*10.05%*
	6	31.73%	*8.26%*	*22.25%*	*1.23%*	*68.27%*	75 536	*10.86%*
	7	29.85%	*8.52%*	*20.02%*	*1.32%*	*70.15%*	63 401	*9.11%*
	8	31.11%	*7.72%*	*22.04%*	*1.36%*	*68.89%*	67 254	*9.67%*
	9	31.47%	*7.51%*	*22.42%*	*1.54%*	*68.53%*	72 074	*10.36%*
	10 Strong offer	29.52%	*6.59%*	*21.41%*	*1.52%*	*70.48%*	69 206	*9.95%*

Neither the GP nor the gynaecologist performed CUS in approximately 1.3% of the patients. This population was not included in the multivariate analyses, as CUS was probably performed within special programmes or by midwives.

### Distribution of access to CUS care according to health professionals

CUS was mainly performed by gynaecologists (67.9%), followed by GPs (27.6%) and finally other health professionals (4.5%). The proportion of CUS performed by gynaecologists decreased among women aged 35 years and older, with a very clear decrease after 50 years of age. Conversely, the proportion of women receiving CUS by a GP was relatively stable, and CMU-C beneficiaries always had a lower proportion of CUS performed by gynaecologists than non-beneficiaries, regardless of their EDI level. For non-CMU-C beneficiaries, we observed a regular decrease in the frequency of CUS as their level of deprivation, as measured by the EDI, increased. The availability of gynaecological care (APL) increased the frequency of CUS performed by gynaecologists but did not change the proportion of CUS performed by GPs. Receiving CUS in a gynaecologist's office was strongly associated with living in urban and Toulouse areas. On the other hand, no geographical variations were observed in CUS performed by GPs.

### Patient characteristics receiving CUS: multinomial multivariate analysis (Table 2)

**Table 2:** Multinomial logistic regression: CUS performed by GP, Gynaecologist vs. no CUS^b^

		**Unadjusted**				**Adjusted** ^a^			
		**CUS performed by GP/CUS not performed**		**CUS performed by gynaecologist/not performed**		**CUS performed by GP/not performed**		**CUS performed by gynaecologist/not performed**	
		OR	*p*¤	OR	*p*¤	Adj OR	*p*¤	Adj OR	*p*¤
**Age** **[5 years]**	^b^25-29	1.00		1.00		1.00		1.00	
	30–34	1.13	< *0.001*	1.10	< *0.001*	1.11	< *0.001*	1.10	< *0.001*
	35–39	1.27	< *0.001*	1.04	*0.001*	1.23	< *0.001*	1.03	*0.005*
	40–44	1.33	< *0.001*	0.98	*0.045*	1.29	< *0.001*	0.97	*0.031*
	45–49	1.26	< *0.001*	0.93	< *0.001*	1.22	< *0.001*	0.93	< *0.001*
	50–54	1.08	< *0.001*	0.77	< *0.001*	1.05	*0.052*	0.77	< *0.001*
	55–59	0.92	< *0.001*	0.59	< *0.001*	0.90	< *0.001*	0.60	< *0.001*
	60–64	0.84	< *0.001*	0.51	< *0.001*	0.81	< *0.001*	0.51	< *0.001*
**Rural/** **urban areas**	^b^Toulouse	1.00		1.00		1.00		1.00	
	Urban	0.98	*0.050*	0.75	< *0.001*	0.96	*0.027*	0.79	< *0.001*
	Rural	0.92	< *0.001*	0.60	< *0.001*	0.94	< *0.001*	0.73	< *0.001*
**No** **CMU**	^b^EDI 1 privileged	1.00		1.00		1.00		1.00	
	EDI 2	0.98	*0.292*	0.92	< *0.001*	0.98	*0.430*	0.95	< *0.001*
	EDI 3	1.03	*0,519*	0.82	< *0.001*	1.06	*0.060*	0.89	< *0.001*
	EDI 4	0.93	< *0.001*	0.79	< *0.001*	0.96	*0.382*	0.89	< *0.001*
	EDI 5	0.89	< *0.001*	0.75	< *0.001*	0.92	*0.002*	0.82	< *0.001*
	EDI 6	0.86	< *0.001*	0.69	< *0.001*	0.91	< *0.001*	0.82	< *0.001*
	EDI 7	0.83	< *0.001*	0.71	< *0.001*	0.88	< *0.001*	0.83	< *0.001*
	EDI 8	0.83	< *0.001*	0.68	< *0.001*	0.88	< *0.001*	0.80	< *0.001*
	EDI 9	0.76	< *0.001*	0.66	< *0.001*	0.81	< *0.001*	0.75	< *0.001*
	EDI 10 deprived	0.68	< *0.001*	0.58	< *0.001*	0.74	< *0.001*	0.63	< *0.001*
**CMU**	EDI 1 privileged	0.88	*0,363*	0.57	< *0.001*	0.87	*0. 538*	0.53	< *0.001*
	EDI 2	0.70	< *0.001*	0.55	< *0.001*	0.69	< *0.001*	0.51	< *0.001*
	EDI 3	0.89	*0.381*	0.50	< *0.001*	0.89	*0.505*	0.48	< *0.001*
	EDI 4	0.75	< *0.001*	0.50	< *0.001*	0.75	< *0.001*	0.51	< *0.001*
	EDI 5	0.83	*0.013*	0.50	< *0.001*	0.84	*0.050*	0.49	< *0.001*
	EDI 6	0.71	< *0.001*	0.46	< *0.001*	0.72	< *0.001*	0.48	< *0.001*
	EDI 7	0.68	< *0.001*	0.48	< *0.001*	0.69	< *0.001*	0.51	< *0.001*
	EDI 8	0.77	< *0.001*	0.50	< *0.001*	0.79	< *0.001*	0.52	< *0.001*
	EDI 9	0.69	< *0.001*	0.47	< *0.001*	0.72	< *0.001*	0.48	< *0.001*
	EDI 10 deprived	0.71	< *0.001*	0.49	< *0.001*	0.74	< *0.001*	0.47	< *0.001*
**APL Gyn**	^b^1 Low offer	1.00		1.00		1.00		1.00	
	2	1.14	< *0.001*	1.21	< *0.001*	1.10	< *0.001*	1.11	< *0.001*
	3	1.14	< *0.001*	1.33	< *0.001*	1.07	*0.008*	1.13	< *0.001*
	4	1.19	< *0.001*	1.48	< *0.001*	1.11	< *0.001*	1.24	< *0.001*
	5	1.29	< *0.001*	1.69	< *0.001*	1.15	< *0.001*	1.25	< *0.001*
	6	1.21	< *0.001*	1.77	< *0.001*	1.06	*0.046*	1.29	< *0.001*
	7	1.22	< *0.001*	1.55	< *0.001*	1.14	< *0.001*	1.27	< *0.001*
	8	1.12	< *0.001*	1.74	< *0.001*	1.03	*0.418*	1.38	< *0.001*
	9	1.10	< *0.001*	1.78	< *0.001*	1.04	*0.427*	1.30	< *0.001*
	10 Strong offer	0.94	*0.002*	1.65	< *0.001*	0.92	*0.012*	1.23	< *0.001*

^a^adjusted for all factors: age, geographical area, gynaecological offer, and deprivation according to the CMU-C; for women aged 25 to 64 years according to their characteristics

^b^Reference category

¤ *p* values with Holm correction

We compared patients who had received CUS via a GP or a gynaecologist with women who did not receive CUS. After adjustment for age, geographical area, gynaecological services, and deprivation (EDI by CMU), we observed a decrease in the incidence of CUS with age. This decrease occurred beginning at the age of 35 for gynaecologists and after 45 for GPs. There was also a greater decrease in the realization of CUS by gynaecologists outside of Toulouse (OR = 0.79 in other urban areas versus OR = 0.73 for rural areas). The variations were smaller for GPs (OR = 0.96 and OR = 0.94, respectively).

We observed a strong impact of deprivation (as shown by EDI) on the realization of CUS by gynaecologists (OR varying from 1 to 0.63 between the lowest and the most deprived deciles in women who were not CMU-C beneficiaries). Among CMU-C beneficiaries, those receiving CUS from a gynaecologist had an even lower probability (OR varying from 0.53 to 0.47). The difference was less pronounced for CUS performed by a GP, with ORs varying from 1 to 0.74 for CMU-C non-beneficiaries and from 0.87 to 0.74 for CMU-C beneficiaries.

The amount of gynaecological care available increased the amount of CUS performed by gynaecologists. The availability of care had an impact on the realization of CUS by GPs, but not by gynaecologists.

**Fig. 2:**
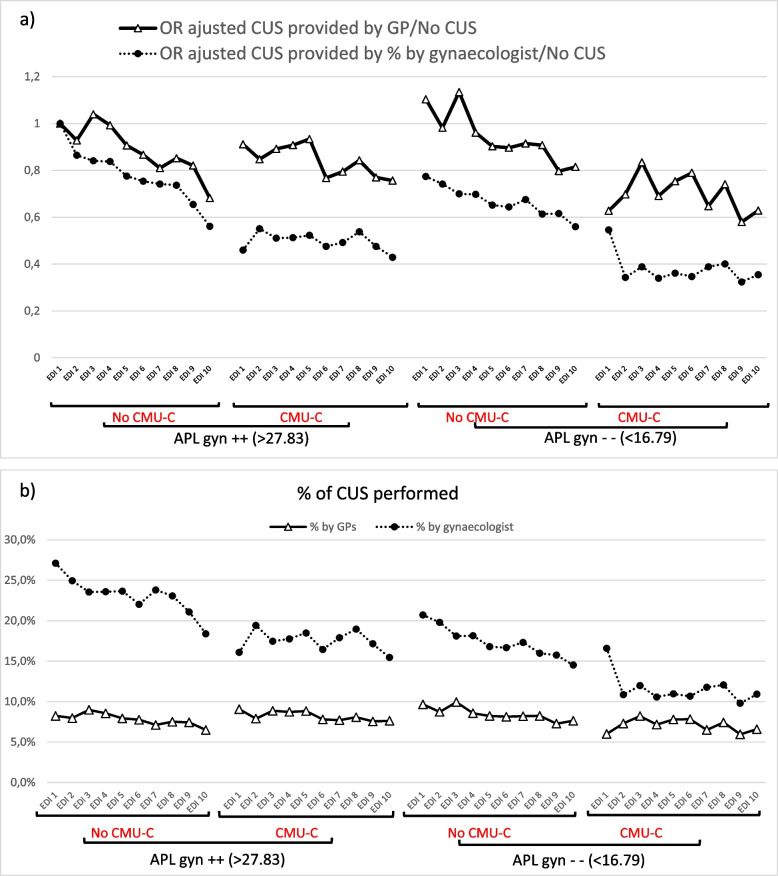
**a** Proportion of CUS performed by GPs and gynaecologists according to their availability. **b** Realization of CUS by GPs and gynaecologists vs. no CUS according to the availability of gynaecological signs*. Legend: ^¤^ Low availability of gynaecologic care = APL Gyn < 16, 79. ^¤¤^ High availability of gynaecologic care = APL Gyn > 27.83. *Multivariate logistic regression adjusted for age and geographical area

To study the impact of the supply of care on the choice of practitioner performing CUS, we differentiated the geographical areas corresponding to two terciles, one with the lowest availability of care (APL gyn < 16.79) and the other with the highest availability (APL gyn > 27.83), to compare the behaviour of two populations exposed to different care services (Fig. 2). When the availability of CUS was low, the activity of the GPs did not compensate for the decrease in the amount of CUS performed. However, the percentage of patients treated by gynaecologists was still greater than that of the other patients but fell from 21.8% to 16.6% (versus 7.6% and 8.2% for GPs) (Fig. 2-a and Appendix).

The association between gynaecologists or GPs performing CUS and the level of deprivation varied according to the availability of gynaecologic care (Fig. 2-b). The amount of CUS performed by gynaecologists decreased when patients were deprived (EDI > 5 and CMU-C), particularly when the availability of gynaecological care was low (OR = 0.38). The amount of CUS performed by GPs was less influenced by deprivation. However, the availability of gynaecological care was low, and the amount of CUS performed by GPs for patients receiving CMU-C and an EDI > 5 also decreased (OR = 0.70).

## Discussion

This retrospective observational study showed that almost 30% of women had a history of CUS within the year, which seemed consistent with the French recommendation of a smear every 3 years, corresponding 33% per year. However, the period of observation was restricted to a single year, which complicated the interpretation of these results, with some women performing more smears than recommended.

Two thirds of the procedures were carried out by gynaecologists, and one third was carried out by GPs. The socioeconomic and geographical characteristics of the patients in this study influenced the amount of cervical cancer screening that they received. Deprivation decreased the screening rate. Moreover, accessing professionals who perform CUS was more difficult for women in rural areas, as the incidence of CUS strongly decreases. The screenings performed by GPs were more homogeneous and penalised deprived patients to a lesser extent even if they represented a minority of the CUS patients.

In France, the proportion of CUS performed by GPs increased from approximately 10% in 1998 [20] to 27% in 2012 in our study. Nevertheless, CUS was still performed by gynaecologists. Patients are generally referred to a physician if additional examinations, such as colposcopy, are needed. Although the incidence of cervical cancer increases from the age of 30 onwards to a maximum of approximately 17 cases per 100,000 women between the ages of 45 and 55 years [21, 22], increasingly fewer CUS procedures are performed after the age of 35 years [23]. Older women are consulting gynaecologists less often because CUS with GPs is more regular over time. GPs probably provide better follow-up information over the years since they are also referral doctors for other pathologies. A US study has shown that access to care can vary according to geographical area and health care availability [24]. In our study, women in rural areas received less CUS, notably because the incidence of CUS performed by gynaecologists was lower there. Conversely, the activity of GPs was relatively stable or was even slightly increasing in rural areas.

We used two indicators to assess socioeconomic level: the CMU-C, which is an individual marker of a very low economic level [13]; and deprivation, a multifactorial concept here measured by the ecological index EDI, reflecting the composition of the population and the place of residence. These two socioeconomic dimensions had an impact on the frequency of CUS. Women receiving CMU-C underwent less CUS than did the others, and the frequency of CUS among them was lower the more they lived in deprived areas. An even sharper gradient in deprivation was observed for women without CMU-C. Gynaecologists' practices are more socially unequal than those of GPs.

By covering all costs, the CMU-C is both a marker of major individual precariousness and a mechanism for compensating for inequalities in access to care. Based on our results, this compensatory mechanism did not work in the same way depending on the doctor consulted. In France, in 2012, 93% of general medicine procedures were performed without the need for a supplementary fee, as compared to 64% for the procedures performed by technical specialists, including gynaecologists [25, 26]. In addition, practitioners who charge a supplementary fee more frequently refuse patients who are CMU-C beneficiaries [27], thus increasing the difficulty of accessing gynaecologists for deprived patients. Unfortunately, there is a social gradient in the risk of cervical cancer [28], which has been confirmed in France [29]. In our study, the more deprived patients were the less likely to receive CUS. This phenomenon was not consistent with the foundation of disease prevention, which should focus on vulnerable groups. From this public health perspective, it is regrettable that GPs are less active than gynaecologists because they have a more egalitarian attitude regarding socioeconomic status.

Regarding the impact of the availability of gynaecologists, we showed that women in areas where gynaecological care was less common had stable access to GPs for CUS (Fig. 2a), even though the availability of general practice was lower. When the availability of care in an area decreases, the last option considered may be general practice. However, GPs tended to behave more unequally, particularly between patients with and without CMU-C (Fig. 2b). Improving access for all professionals—gynaecologists and GPs—is necessary to limit these inequalities. In France, gynaecologists are perceived by women as the doctor of choice for gynaecological follow-up. However, neither their training, their number, nor their geographical distribution allow them to fulfil this role correctly, as observed in our study. In addition, a 62% decrease in the number of primary care gynaecologists over 13 years versus 8% for GPs [30] will make access to a gynaecologist increasingly difficult. An organisational rethinking should be required when CUS is performed by GPs or midwives and when the gynaecologist is positioned as a referral doctor. A study in France showed that when availability of gynaecological care decreased, GP consultations included an increasing frequency of gynaecological procedures such as speculum examination or CUS (52% and 39%, respectively, versus 41% and 29%, respectively, in the event of a high gynaecological APL) [31].

General practice is concerned with all nine levels of care [32]. Like general practitioners, midwives are professionals involved in primary care and the management of women's health. Primary care should be accessible, continuous, comprehensive and coordinated [1]. In the United States, the presence of a GP in the immediate proximity of patients has reduced inequalities in care, even if inequalities in access have persisted [5, 6, 33]. Recognition of the GP as a referral doctor for deprived populations requires awareness and training of primary care practitioners. Financial incentives for health professionals taking care of disadvantaged patients are not sufficient to improve access to care [34]. Midwives have been carrying out CUS screening since 2009; even if their participation was very small in 2012, they are increasingly involved in carrying out smear screening. GPs and midwives need specific training in the specific problems faced by deprived populations. This training must involve primary care gynaecologists who are the professionals who carry out most of the CUS, are pivotal in care in gynaecology and are the most able to improve access to smears. Refocusing the organisation of the health system around primary care may reduce health inequalities [35]. An in-depth rethinking may be necessary to facilitate the organisation of care, so that the health needs of the deprived patients can be met satisfactorily once they have been screened.

The main strengths of our study were methodological. First, all patients were recruited from the Midi-Pyrénées region without any medical exclusion criteria. In total, 13% of the population was not covered by the data source, represented by special schemes (e.g., notaries, the French National Railway Company) and people not affiliated with social security. This rate was quite low and was not expected to influence our results significantly. Second, the population included all professions (employed, self-employed, unemployed) and socioeconomic levels. Third, cervical cancer screening was performed by using factual and non-declarative data to avoid measurement bias. The data came exclusively from CUS invoking and were almost exhaustive.

This study has several limitations. First, we arbitrarily assigned the realization of CUS to a type of practitioner, because the information on the practitioner who performed CUS was not available in the data source. We considered that when a gynaecologist performed a consultation, a CUS was done. However, patients may be referred by a GP to a gynaecologist when complex a CUS is expected. This hypothesis may have weakened the statistical power of this work by homogenising the populations studied. Similarly, some smears were performed by midwives. Although rare, this practice could not be included in our study. Second, we measured the percentage of women who underwent CUS for only one year. Due to the technical and legal complexity of creating a database combining reimbursement data and socio-demographic data, we have only been able to collect data for one year. Women who underwent CUS were 29.1% in 2012, which corresponded to a screening rate of 87.3% over 3 years. However, between 2010 and 2013, the incidence of CUS was between 61 and 70% in the same region [36]. It might be explained by an overconsumption of CUS, with regard to the recommendations, by a part of the population.

## Conclusions

In our cohort, 29% of women underwent CUS in 2012. However, social inequalities in access to CUS seemed to persist. Women living in rural or deprived areas and those lacking health care services had a much lower uptake of CUS, yet they constituted the population that needs CUS the most. Gynaecologists performed the two thirds of CUS procedures, but their practice seemed associated with social inequalities. GPs seemed to have more homogeneous practices regarding the socioeconomic and geographical characteristics of patients, with the result that deprived people seemed less penalised by GPs, but still receive few CUS.

Involvement of GPs may improve cervical screening equality in France. The organisation of health systems around primary care should allow a better access to care and a better care for the deprived population. The specific needs of these populations must become a priority for medical training, particularly in terms of screening and prevention. Raising GPs' and midwives’ awareness of identifying vulnerable populations and optimising their access to GPs would improve their access to health care in general. In France, this agenda will require an in-depth rethinking involving the entire medical profession, including specialists, with a specific organisation for vulnerable patients.

## Supplementary Information

Supplementary Material 1.

## Data Availability

The data that support the findings of this study are available from CERPOP (Toulouse Inserm team, contact Cyrille Delpierre cyrille.delpierre@inserm.fr), but restrictions apply to the availability of these data, which were used under licence for the current study and are not publicly available. However, the data are available from the authors upon reasonable request and with permission of the French National Health Data System (SNDS) and from the ‘Commission Nationale Informatique et Libertés’ (CNIL). This process can be performed on the SNDS website (https://www.snds.gouv.fr/SNDS/Processus-d-acces-aux-donnees).
