# Supplementary material for: General practitioners may improve cervical screening equality in France
Source: BMC Public Health. 2024 Oct 9;24:2748. doi: 10.1186/s12889-024-18942-8 (PMC11462826; doi:10.1186/s12889-024-18942-8)
Supplement: Supplementary file 1 — Supplementary Material 1. [file 12889_2024_18942_MOESM1_ESM.docx]

# Appendix:

|  | | | **Description** | | | | **Multinomial logistic regression** | | | |
| --- | --- | --- | --- | --- | --- | --- | --- | --- | --- | --- |
|  |  |  | **CUS by GP** | **CUS by Gynaeco** | **No CUS** | ***Total*** | **CUS by GP / CUS not performed** | | **CUS by gynaecologist / CUS not performed** | |
|  |  |  | *7.90%* | *19.19%* | *72.90%* | *Nb =* 457,623 (%) | Adjust OR | *p* | Adjust OR | *p* |
| **Age**  **[5 years]** | | *25-29 | 7.05% | 22.09% | 70.86% | 56 636 (12.4%) | 1.00 |  | 1.00 |  |
|  |  | 30-34 | 7.62% | 23.48% | 68.90% | 59 053 (12.9%) | 1.10 | *<0.001* | 1.10 | 0.015 |
|  |  | 35-39 | 8.46% | 22.14% | 69.40% | 54 969 (12.0%) | 1.20 | *<0.001* | 1.04 | 0.022 |
|  |  | 40-44 | 9.17% | 20.63% | 70.19% | 58 892 (12.9%) | 1.28 | *<0.001* | 0.97 | *<0.001* |
|  |  | 45-49 | 8.83% | 20.07% | 71.10% | 60 313 (13.2%) | 1.22 | *<0.001* | 0.93 | *<0.001* |
|  |  | 50-54 | 8.04% | 17.62% | 74.34% | 57 794 (12.6%) | 1.06 | *0.007* | 0.78 | *<0.001* |
|  |  | 55-59 | 7.12% | 14.42% | 78.46% | 55 515 (12.1%) | 0.89 | *<0.001* | 0.61 | *<0.001* |
|  |  | 60-64 | 6.78% | 12.57% | 80.65% | 54 451 (11.9%) | 0.82 | *<0.001* | 0.52 | 0.015 |
| **Geo zone** | | *Toulouse | 7.61% | 22.88% | 69.51% | 189 984 (41.5%) | 1.00 |  | 1.00 |  |
|  |  | Urban | 8.35% | 18.34% | 73.31% | 105 746 (21.1%) | 1.03 | *0.097* | 0.81 | *<0.001* |
|  |  | Rural | 7.96% | 15.42% | 76.62% | 161 893 (35.4%) | 0.93 | *<0.001* | 0.72 | *<0.001* |
| **APL > 27.83**  **(high)** | **No CMU** | *EDI 1 | 8.23% | 27.11% | 64.66% | 18 750 *(4.1%)* | 1.00 |  | 1.00 |  |
|  |  | EDI 2 | 7.96% | 24.94% | 67.11% | 15 396 *(3.4%)* | 0.93 | *0.064* | 0.87 | *<0.001* |
|  |  | EDI 3 | 8.97% | 23.54% | 67.49% | 14 040 *(3.1%)* | 1.04 | 0.333 | 0.84 | *<0.001* |
|  |  | EDI 4 | 8.55% | 23.57% | 67.88% | 14 715 *(3.2%)* | 0.99 | 0.866 | 0.84 | *<0.001* |
|  |  | EDI 5 | 7.92% | 23.65% | 68.43% | 20 151 *(4.4%)* | 0.91 | 0.010 | 0.78 | *<0.001* |
|  |  | EDI 6 | 7.75% | 22.02% | 70.23% | 15 070 *(3.3%)* | 0.87 | 0.001 | 0.75 | *<0.001* |
|  |  | EDI 7 | 7.11% | 23.78% | 69.11% | 17 086 *(3.7%)* | 0.81 | *<0.001* | 0.74 | *<0.001* |
|  |  | EDI 8 | 7.50% | 23.07% | 69.43% | 16 924 *(3.7%)* | 0.85 | *<0.001* | 0.74 | *<0.001* |
|  |  | EDI 9 | 7.43% | 21.09% | 71.49% | 30 725 *(6.7%)* | 0.82 | *<0.001* | 0.66 | *<0.001* |
|  |  | EDI 10 | 6.48% | 18.37% | 75.15% | 40 307 *(8.8%)* | 0.68 | *<0.001* | 0.56 | *<0.001* |
|  | **CMU** | EDI 1 | 9.06% | 16.08% | 74.86% | 541 *(0.1%)* | 0.91 | 0.550 | 0.46 | *<0.001* |
|  |  | EDI 2 | 7.89% | 19.40% | 72.70% | 773 *(0.2%)* | 0.85 | 0.227 | 0.55 | *<0.001* |
|  |  | EDI 3 | 8.85% | 17.45% | 73.70% | 768 *(0.2%)* | 0.89 | 0.388 | 0.51 | *<0.001* |
|  |  | EDI 4 | 8.72% | 17.75% | 73.53% | 952 *(0.2%)* | 0.91 | 0.417 | 0.51 | *<0.001* |
|  |  | EDI 5 | 8.82% | 18.48% | 72.70% | 1 564 *(0.3%)* | 0.93 | 0.468 | 0.52 | *<0.001* |
|  |  | EDI 6 | 7.80% | 16.44% | 75.76% | 1 411 *(0.3%)* | 0.77 | 0.012 | 0.48 | *<0.001* |
|  |  | EDI 7 | 7.70% | 17.88% | 74.41% | 1 493 *(0.3%)* | 0.80 | 0.024 | 0.49 | *<0.001* |
|  |  | EDI 8 | 8.07% | 18.95% | 72.98% | 1 847 *(0.4%)* | 0.84 | 0.060 | 0.54 | *<0.001* |
|  |  | EDI 9 | 7.55% | 17.14% | 75.32% | 4 201 *(0.9%)* | 0.77 | *<0.001* | 0.48 | *<0.001* |
|  |  | EDI 10 | 7.62% | 15.45% | 76.94% | 11 763 *(2.6%)* | 0.76 | *<0.001* | 0.43 | *<0.001* |
| **APL < 16.79**  **(low)** | **No CMU** | EDI 1 | 9.65% | 20.70% | 69.65% | 8 747 *(1.9%)* | 1.10 | 0.031 | 0.77 | *<0.001* |
|  |  | EDI 2 | 8.72% | 19.78% | 71.50% | 14 117 *(3.1%)* | 0.98 | 0.662 | 0.74 | *<0.001* |
|  |  | EDI 3 | 9.93% | 18.09% | 71.97% | 16 309 *(3.6%)* | 1.14 | 0.001 | 0.70 | *<0.001* |
|  |  | EDI 4 | 8.55% | 18.14% | 73.31% | 19 088 *(4.2%)* | 0.96 | 0.305 | 0.70 | *<0.001* |
|  |  | EDI 5 | 8.22% | 16.79% | 74.99% | 21 259 *(4.7%)* | 0.90 | 0.008 | 0.65 | *<0.001* |
|  |  | EDI 6 | 8.13% | 16.66% | 75.21% | 30 852 *(6.7%)* | 0.90 | 0.003 | 0.64 | *<0.001* |
|  |  | EDI 7 | 8.19% | 17.31% | 74.50% | 28 281 *(6.2%)* | 0.92 | 0.016 | 0.68 | *<0.001* |
|  |  | EDI 8 | 8.23% | 15.98% | 75.80% | 28 962 *(6.3%)* | 0.91 | 0.009 | 0.61 | *<0.001* |
|  |  | EDI 9 | 7.26% | 15.74% | 77.00% | 27 857 *(6.1%)* | 0.80 | *<0.001* | 0.62 | *<0.001* |
|  |  | EDI 10 | 7.63% | 14.51% | 77.86% | 18 112 *(4.0%)* | 0.82 | *<0.001* | 0.56 | *<0.001* |
|  | **CMU** | EDI 1 | 5.99% | 16.59% | 77.42% | 217 *(0.1%)* | 0.63 | 0.107 | 0.55 | 0.001 |
|  |  | EDI 2 | 7.30% | 10.85% | 81.85% | 507 *(0.1%)* | 0.70 | 0.040 | 0.34 | *<0.001* |
|  |  | EDI 3 | 8.20% | 11.97% | 79.84% | 610 *(0.3%)* | 0.84 | 0.231 | 0.39 | *<0.001* |
|  |  | EDI 4 | 7.16% | 10.57% | 82.27% | 880 *(0.1%)* | 0.69 | 0.006 | 0.34 | *<0.001* |
|  |  | EDI 5 | 7.78% | 10.95% | 81.27% | 1 105 *(0.2%)* | 0.75 | 0.016 | 0.36 | *<0.001* |
|  |  | EDI 6 | 7.83% | 10.66% | 81.51% | 1 801 *(0.4%)* | 0.79 | 0.010 | 0.35 | *<0.001* |
|  |  | EDI 7 | 6.49% | 11.76% | 81.75% | 1 973 *(0.4%)* | 0.65 | *<0.001* | 0.39 | *<0.001* |
|  |  | EDI 8 | 7.42% | 12.05% | 80.53% | 2 398 *(0.5%)* | 0.74 | *<0.001* | 0.40 | *<0.001* |
|  |  | EDI 9 | 5.95% | 9.79% | 84.26% | 2 973 *(0.7%)* | 0.58 | *<0.001* | 0.32 | *<0.001* |
|  |  | EDI 10 | 6.58% | 10.91% | 82.50% | 3 098 *(0.7%)* | 0.63 | *<0.001* | 0.36 | *<0.001* |

* Reference category

High availability of gynaecologic care = APL Gyn > 27.83 . Low availability of gynaecologic care = APL Gyn < 16.79

## Appendix 1: CUS performed by GP. by gynaecologist vs. no CUS in areas with **a low and high availability of gynaecological care (APL)**. Description of population and multinomial logistic regression.
